# Recurrent Hepatic Encephalopathy After Abdominal Surgery in a Non-Cirrhotic Patient: A Case Report

**DOI:** 10.3390/reports9020112

**Published:** 2026-04-04

**Authors:** Sebastiano Ziola, Giuseppe Cittadini, Andrea Pasta, Sara Labanca, Giulia Pieri, Simona Marenco, Edoardo G. Giannini

**Affiliations:** 1 Gastroenterology Unit, Department of Internal Medicine, University of Genoa, 16132 Genoa, Italy; 2 Azienda Ospedaliera Metropolitana IRCCS Policlinico San Martino, Largo R. Benzi n.10, 16132 Genoa, Italy; 3 Unit of Diagnostic and Interventional Radiology, Azienda Ospedaliera Metropolitana IRCCS Policlinico San Martino, Largo R. Benzi n.10, 16132 Genoa, Italy

**Keywords:** pancreatic adenocarcinoma, portosystemic shunt, rifaximin, case report

## Abstract

Background and Clinical Significance: Hepatic encephalopathy (HE) is a reversible brain dysfunction typically associated with cirrhosis and portal hypertension. In these patients, portosystemic shunts allow ammonia and other toxins to bypass hepatic metabolism, leading to neurological symptoms. However, HE can also occur in non-cirrhotic patients through congenital shunts or, less commonly, through iatrogenic shunts following abdominal trauma or surgery. This case is clinically significant as it illustrates a rare presentation of recurrent HE caused by a de novo portosystemic shunt following major abdominal surgery in a patient without underlying liver disease. Case Presentation: A 76-year-old male was admitted with confusion, lethargy, and flapping tremors. His medical history included a total pancreatectomy for pancreatic adenocarcinoma six months prior. Laboratory tests revealed hyperammonemia and altered liver enzymes likely related to ongoing chemotherapy, but no signs of hepatic insufficiency or cirrhosis. A review of recent CT imaging identified a new portosystemic shunt between the portal territory and the azygous vein that was absent prior to his pancreatectomy. This iatrogenic shunt likely formed via the re-vascularization of vestigial vessels following surgical de-vascularization. The patient was successfully managed with lactulose and rifaximin. At 3-month follow-up, no further HE episodes had occurred. Conclusions: This case highlights that HE should be considered in patients without cirrhosis presenting with altered mental status and hyperammonemia, especially following abdominal surgery. It underscores the importance of a multidisciplinary approach and meticulous re-evaluation of imaging to identify iatrogenic vascular shunts that may be amenable to medical or interventional management.

## 1. Introduction and Clinical Significance

Hepatic encephalopathy (HE) is defined as a reversible brain dysfunction caused by liver insufficiency and/or portal-systemic shunting. It can manifest as a spectrum of neurological or psychiatric abnormalities ranging from subclinical alterations to coma and generally occurs in patients with cirrhosis and end-stage liver disease [1,2]. Non-cirrhosis-related HE is less frequently encountered and is most commonly due to the presence of spontaneous portosystemic shunts [3]. In these cases, the portal blood bypasses the liver and proceeds straight into the venous systemic circulation leading to the accumulation of ammonia that is responsible for the clinical manifestations of HE [3]. Portosystemic shunts not only occur in patients with cirrhosis but also can form congenitally, and after abdominal trauma or surgery [1].

We present the case of a non-cirrhotic patient with recurrent episodes of HE due to the iatrogenic creation of a portosystemic shunt after major abdominal surgery.

## 2. Case Presentation

A 76-year-old male patient was admitted to the Emergency Department of our hospital with confusion and lethargy. At the time of admission, the patient was on chemotherapy (gemcitabine and abraxane every other week) for pancreatic ductal adenocarcinoma following total pancreatectomy 6 months prior. Patient’s additional medical/surgical history included right nephrectomy for clear-cell carcinoma (2010) and prostatic adenocarcinoma treated with radical prostatectomy (2016).

On admission, no clinical signs of stroke were found during physical examination, and brain computed tomography (CT) scan was negative for acute vascular events and metastases. The patient appeared confused, disoriented in terms of time and place, and presented flapping tremors. Blood tests showed moderate anemia (Hgb 99 g/L), minimal thrombocytopenia (133 × 10^9^/L), normal coagulation tests, moderately decreased renal function (eGFR 53 mL/min/1.73 m^2^), no alteration of electrolytes, normal glucose level, altered liver tests (aspartate aminotransferase 180 U/L, alanine aminotransferase 80 U/L, alkaline phosphatase 1018 U/L; gammaglutamlyltranspeptidase 1598 U/L, total bilirubin 1.44 mg/dL) and hyperammonemia (137 umol/L), with no signs of hepatic insufficiency. A similar episode had occurred a few weeks earlier, with spontaneous, rapid recovery without any therapeutic intervention.

**Figure 1:**
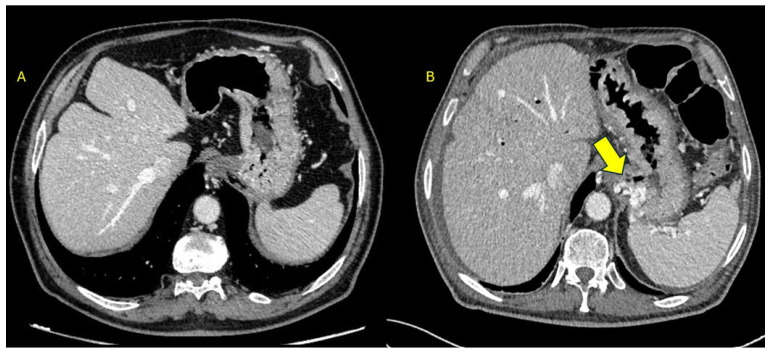
Sagittal imaging. Comparison between pre-operative (**A**) and post-operative (**B**) CT scans. The arrow indicates the iatrogenic portosystemic shunt between the portal territory and the azygous vein.

**Figure 2:**
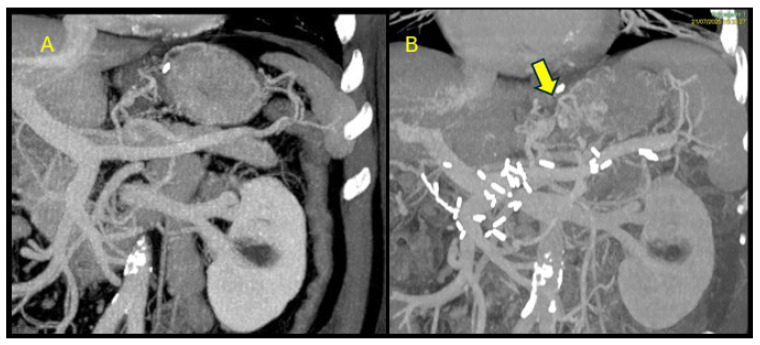
Coronal imaging. Comparison between pre-operative (**A**) and post-operative (**B**) CT scans. The arrow indicates the iatrogenic portosystemic shunt between the portal territory and the azygous vein.

The patient was transferred to our unit and treated with lactulose and rifaximin, with a prompt recovery of the state of consciousness and normalization of ammonia levels. Since there were no clinical signs of chronic liver disease or portal hypertension, we reviewed the most recent whole-body CT images performed during the oncological follow-up three weeks before admission. Imaging review identified the presence of a portosystemic shunt between the portal territory and the azygous vein that was absent before pancreatectomy (Figure 1A and Figure 2A) and that we considered the most likely explanation for the recurrent episodes of hepatic encephalopathy in the absence of cirrhosis.

This finding was likely due to the re-vascularization of some vestigial vessels following de-vascularization of the pancreatic territory following pancreatectomy and vessel clipping (Figure 1B and Figure 2B).

After oncology consultation and an assessment of the patient’s preference, we opted for medical management of HE with chronic lactulose and rifaximin rather than interventional shunt embolization. At the 3-month follow up visit, no other hepatic encephalopathy episodes had occurred.

## 3. Discussion

HE is generally considered one of the most severe and worrisome complications of end-stage liver disease and, despite improvement in medical management, still represents a tough challenge for clinicians in the case of recurrent, or refractory, manifestations.

HE usually affects patients with cirrhosis and portal hypertension (type C-HE), in whom the presence of portosystemic shunts leads to brain accumulation of ammonia, which in turn is responsible for the onset of symptoms such as confusion, disorientation, lethargy, and flapping tremor. Though less common, HE can be secondary to hyperammonemia in the absence of any hepatic dysfunction (type B-HE), in which portal-systemic shunting is the cause of HE development without an underlying liver disease. In these cases, a correct diagnosis is not always easy to reach, as patients with no history of liver disease symptoms can be frequently misdiagnosed with neurologic or psychiatric disorders [4,5]. Quite a few cases are reported in the literature regarding the development of portosystemic shunts following pancreatic surgery, and in at least one of these cases the development of HE was secondary to the enlargement of a pre-existing splenorenal shunt [6,7].

Portosystemic shunts not associated with liver cirrhosis and portal hypertension are rare; in most cases, they have a congenital origin and are due to abnormal development, or involution, of fetal vasculature, while less commonly they can be a consequence of abdominal trauma or prior surgery [6,7,8,9]. Indeed, our patient had previously undergone three abdominal surgeries, including total pancreatectomy and nephrectomy. In our case, the presence of altered liver tests might have further misled the diagnostic pathway, and we ruled out the most common causes of chronic liver disease via the patient’s history, laboratory tests, and imaging, and attributed their origin to chemotherapy as they appeared after its beginning. Moreover, a review of abdominal scans performed during oncological follow-up led to the identification of a shunt that had not previously been described, emphasizing the role of disease knowledge and of dedicated radiologists.

Lastly, from a therapeutic point of view, on admission, our patient presented hyperammonemia that subsided after laxative therapy, with resolution of symptoms. While cases of HE due to congenital or iatrogenic portosystemic shunts have previously been described, and some successfully treated with embolization of the vessel, in this case we decided to proceed with pharmacological management alone due to several factors such as the patient’s performance status (Eastern Cooperative Oncology Group score 2), previous surgery with devascularization that prevented safe embolization, cancer-related prognosis, preference, and rapid recovery with assumption of lactulose and rifaximin [1,7,9,10]. Furthermore, the patient presented with marked sarcopenia, and although the presence of sarcopenia is a well-known factor associated with a higher risk of HE occurrence and recurrence in patients with cirrhosis, we also feel that in this case muscle loss might have decreased the potential to scavenge circulating ammonia [11]. We are aware that rifaximin is indicated as a therapeutic add-on to lactulose following primary failure; however, due to the frailty of the patient, we preferred to safeguard the patient rather than waiting for new episodes. Despite the follow-up still being immature, secondary prophylaxis with both lactulose and rifaximin was associated with no recurrent episodes of HE.

## 4. Conclusions

This report highlights the importance of thinking outside the box, as HE can also occur in patients with no history of liver disease, and the presence of altered liver tests should not be considered, a priori, a sign of chronic liver disease since there may be other concurrent factors leading to liver injury without compromising liver detoxifying functions [12]. Furthermore, it underscores the need to reassess previous, apparently negative examinations, as their re-evaluation with dedicated queries, as in this case, might lead to the identification of findings responsible for clinical manifestations and to the modification of diagnosis and prognosis.

## Data Availability

The original data presented in this study are available on reasonable request from the corresponding author. The data are not publicly available due to privacy concerns.

## Abbreviations

The following abbreviations are used in this manuscript:

HE	hepatic encephalopathy
CT	computed tomography
